# GDF-15 and Hepcidin Levels in Nonanemic Patients with Impaired Glucose Tolerance

**DOI:** 10.1155/2016/1240843

**Published:** 2016-08-25

**Authors:** Mehmet Muhittin Yalcin, Alev Eroglu Altinova, Mujde Akturk, Ozlem Gulbahar, Emre Arslan, Damla Ors Sendogan, Ilhan Yetkin, Fusun Balos Toruner

**Affiliations:** ^1^Department of Endocrinology and Metabolism, Gazi University Faculty of Medicine, 06560 Ankara, Turkey; ^2^Department of Biochemistry, Gazi University Faculty of Medicine, 06560 Ankara, Turkey; ^3^Department of Internal Medicine, Gazi University Faculty of Medicine, 06560 Ankara, Turkey

## Abstract

*Aims*. Growth Differentiation Factor-15 (GDF-15) has been suggested as one of the regulators of hepcidin, an important regulatory peptide for iron deposition. Current data is conflicting about the relationship between hepcidin and disorders of glucose metabolism. We aimed to investigate serum hepcidin and GDF-15 concentrations and their associations with each other, in nonanemic subjects with impaired glucose tolerance (IGT) in comparison with the nonanemic subjects with normal glucose tolerance (NGT).* Methods*. Thirty-seven subjects with IGT and 32 control subjects with NGT, who were age-, gender-, and body mass index- (BMI-) matched, were included in the study.* Results*. Serum GDF-15 levels were significantly higher in IGT compared to NGT. There were no differences in hepcidin, interleukin-6, and high sensitive C-reactive protein levels between the groups. We found a positive correlation between GDF-15 and hepcidin levels. There were also positive correlations between GDF-15 and age, uric acid, creatinine, and area under the curve for glucose (AUC-G). Hepcidin was correlated positively with ferritin levels. In the multiple regression analysis, GDF-15 concentrations were independently associated with age, uric acid, and AUC-G.* Conclusions*. Impaired glucose tolerance is associated with increased GDF-15 levels even in the absence of anemia, but the levels of hepcidin are not significantly altered in prediabetic state.

## 1. Introduction

High iron concentrations have been suggested to be associated with subclinical inflammation and increased oxidative stress in type 2 diabetes mellitus (T2DM) [1]. Besides, a positive association between fasting glucose and insulin levels and chronic iron deposition has been reported in a population study [2].

Hepcidin is a peptide hormone that regulates iron homeostasis in the body [3]. Hepcidin is synthesized from the hepatocytes and causes negative effect on iron absorption and cellular release via binding to ferroportin [3]. Alterations in hepcidin levels have been reported in chronic diseases with iron deposition [4, 5]. There are a growing number of studies targeting the possible role of hepcidin in the pathogenesis of insulin resistance and related diseases [6–8]. Decreased or unchanged hepcidin concentrations have been found in patients with polycystic ovary and metabolic syndrome [9–11]. In patients with T2DM, some studies have reported decreased circulating hepcidin levels while others showed no change [10, 12, 13]. To the best of our knowledge, there is no data regarding hepcidin concentrations in subjects with impaired glucose tolerance.

Growth Differentiation Factor-15 (GDF-15), which is also known as macrophage inhibitory cytokine 1 (MIC-1), has been suggested as one of the regulators of hepcidin [14]. GDF-15 is an anti-inflammatory cytokine secreted mainly from the macrophages in response to oxidative stress and inflammation [15]. Plasma GDF-15 levels have been found to be positively correlated with abdominal obesity and insulin resistance in obese subjects without diabetes [16]. Also, increasing effect of hyperinsulinemia on circulating GDF-15 concentrations has been shown in a clamp study [17]. Increased GDF-15 levels have been reported in patients with T2DM [18, 19] and high GDF-15 has been suggested to be a compensatory anti-inflammatory mechanism in the development of T2DM [20]. Previous studies on the association between GDF-15 and prediabetes are very few [16, 21, 22].

A strong positive relationship between hepcidin and GDF-15 has been found in anemic patients with T2DM [23]. It has been suggested that anemia may have an effect on the hepcidin and GDF-15 levels [24], but no data is available regarding the hepcidin and GDF-15 levels and their possible relationships in nonanemic subjects with prediabetes. In our point of view, excluding anemic subjects is important to better understand the relationship between hepcidin, GDF-15, and insulin resistance.

Our aim here is to investigate serum hepcidin and GDF-15 concentrations and their associations with each other in nonanemic subjects with impaired glucose tolerance and compare them with the nonanemic subjects with normal glucose tolerance.

## 2. Material and Methods

### 2.1. Subjects

From the individuals who underwent 75-gr oral glucose tolerance test (OGTT) in our Endocrinology Department, 37 subjects with impaired glucose tolerance were selected to participate in the present study. Impaired glucose tolerance was identified as 2-h glucose between 140 and 199 mg/dL. Subjects with fasting blood glucose level ≥ 126 mg/dL were excluded. The subjects with anemia (<12 g/L for women; <13 g/L for men) and low ferritin levels (<20 ng/mL) and subjects taking medications for anemia or subjects with any chronic disease that may influence the iron metabolism were excluded. Other exclusion criteria were acute or chronic infections, malignancies, and chronic hepatic or renal disease.

Thirty-two age-, gender-, and body mass index- (BMI-) matched subjects with normal glucose tolerance according to 75-gr OGTT (fasting blood glucose < 100 mg/dL, 2-h glucose < 140 mg/dL) were included in the study as the control group.

### 2.2. Biochemical Analysis

Eight-hour overnight fasting blood samples were collected from participants in the morning for biochemical evaluation. Complete blood count, insulin, uric acid, creatinine, and ferritin levels were studied. Also, blood samples were collected for the measurement of hepcidin, GDF-15, interleukin-6 (IL-6), and high sensitivity C-reactive protein (hsCRP). These samples were stored in −80°C freezer. Glucose levels were measured at 0, 30, 60, 90, and 120 minutes of 75-gr OGTT. A diet containing 300 gr carbohydrates/day was advised at least for 3 days before the test.

Area under the curve for glucose (AUC-G) was calculated by trapezoidal rule for all participants. The homeostasis model assessment of insulin resistance (HOMA-IR) was calculated as previously published [25]. Estimated glomerular filtration rate was calculated by the four-variable Modification of Diet in Renal Disease (MDRD) equation [26].

Fasting blood glucose, glucose levels from OGTT, uric acid, creatinine levels were measured by Cobas 8000 model Roche autoanalyser with colorimetric methods. Insulin and ferritin levels were measured by electrochemiluminescence method with Cobas 8000 model Roche autoanalyser. Complete blood count was determined by a Beckman/Coulter Model LH 750 Hematology Analyser. Serum hsCRP levels were determined by nephelometric method with BN ProSpec model analyser (Siemens). Serum Hepcidin 25 (bioactive) (DRG, USA), human GDF-15/MIC-1 (BioVendor, Czech Republic), and Human IL-6 Platinum (eBioscience, Austria) were measured by enzyme-linked immunosorbent assay (ELISA) method.

The study was approved by the institutional ethics committee, and informed consent was obtained from all participants included in the study.

### 2.3. Statistical Analysis

Statistical analyses were performed using IBM SPSS Statistics for Windows v21.0 (IBM Corp.). Continuous variables are presented as the median [25th, 75th percentile]. Chi-square test was performed to evaluate the gender differences between the groups. Normality of the distribution was investigated with Shapiro-Wilk's test. The differences between independent groups in terms of numerical variables were examined using the *t*-test or the Mann-Whitney* U *test, according to the provided condition of parametric or nonparametric distribution. Pearson or Spearman correlation analysis was performed to evaluate the relationship between numeric variables. Multiple regression analysis was performed to investigate the factors affecting GDF-15 and hepcidin levels. As GDF-15 levels showed nonparametric distribution, logarithmic transformation was applied to GDF-15 levels before the regression analysis. *p* value of < 0.05 was considered statistically significant.

## 3. Results

Baseline characteristics of the patient and control groups were shown in Table 1. There were no differences in age, gender, BMI, hemoglobin, ferritin, creatinine, and uric acid between the groups (*p* > 0.05). Fasting blood glucose (106.0 [99.50–116.0] versus 92.0 [88.25–97.0] mg/dL, *p* < 0.001), AUC-G (22002.97 ± 2917.88 mg/dL/min versus 14627.0 ± 2932.26 mg/dL/min, *p* < 0.001), and HOMA-IR (3.95 ± 2.21 versus 2.75 ± 1.49, *p* = 0.012) were higher in the patient group than in control group. GDF-15 levels were significantly elevated in subjects with impaired glucose tolerance compared to the subjects with normal glucose tolerance (897.93 [691.57–1616.10] versus 770.36 pg/mL [535.34–1040.0], *p* = 0.026). There were no differences in hepcidin (25.89 ± 15.60 versus 26.94 ± 13.05 ng/dL, *p* > 0.05), IL-6 (1.24 [0.94–1.64] versus 1.07 [0.92–1.57] pg/mL, *p* > 0.05), and hsCRP (0.32 [0.15–0.73] versus 0.20 [0.11–0.48] mg/dL, *p* > 0.05) levels between the groups.

In the whole group, there was a positive correlation between GDF-15 and hepcidin levels (*r* = 0.248, *p* = 0.04) (Table 2). There were also positive correlations between GDF-15 and age (*r* = 0.625, *p* < 0.001), uric acid (*r* = 0.294, *p* < 0.05), creatinine (*r* = 0.298, *p* < 0.05), and AUC-G (*r* = 0.261, *p* < 0.05). Hepcidin was correlated positively with ferritin levels (*r* = 0.449, *p* < 0.001).

Multiple regression analysis consisting of age, uric acid, AUC-G, creatinine, and hepcidin revealed that GD multiple regression analysis consisting of age, uric acid, AUC-G, creatinine, and hepcidin revealed that GDF-15 concentrations were independently associated with age (*β* = 0.533, *p* < 0.01), uric acid (*β* = 0.244, *p* < 0.05), and AUC-G (*β* = 0.206, *p* < 0.05) (*r* ^2^ = 0.486, *p* < 0.001). Ferritin (*β* = 0.464, *p* < 0.01) was found to be significant predictor for hepcidin levels in the multiple regression analysis model including ferritin, gender, fasting blood glucose, and GDF-15 levels (*r* ^2^ = 0.215, *p* < 0.001).

## 4. Discussion

In the present study, we demonstrated that serum GDF-15 concentrations are increased in nonanemic subjects with impaired glucose tolerance compared to subjects with normal glucose tolerance. However, there was no difference in the levels of hepcidin between two groups.

Hepcidin has a key role in the iron metabolism and elevated hepcidin levels were shown to be associated with chronic iron overload, especially chronic disease anemia [5]. Several studies have demonstrated chronic iron overload in patients with diabetes and prediabetes [27]. Regarding the relationship between hepcidin and abnormal glucose metabolism, Sam et al. reported that the patients with T2DM have lower hepcidin levels than age-, gender-, and BMI-matched control subjects [10]. In contrast, Jiang et al. reported elevated hepcidin levels in T2DM compared to control group without diabetes [28]. Controversial results of hepcidin levels in T2DM also exist in the previous studies on metabolic syndrome [6, 29]. Conflicting data about hepcidin levels in the diseases associated with insulin resistance can partly be explained with the strong positive correlation between hepcidin and ferritin levels. Some previous studies which found elevated levels of ferritin in the patient group also found elevated hepcidin levels in the same group [28, 30]. To the best of our knowledge, this is the first study investigating hepcidin levels in nonanemic subjects with impaired glucose tolerance. Further studies focusing on the relationship between hepcidin levels and insulin resistance excluding ferritin effect are needed.

GDF-15 is an anti-inflammatory cytokine which was suggested as one of the regulators of hepcidin. The role of GDF-15 in the abnormal glucose metabolism has started to be investigated recently. Hong et al. [21] reported elevated GDF-15 levels in subjects with impaired fasting glucose. In their study, age and BMI of the patient group were higher than those of control subjects and GDF-15 levels were significantly higher compared to control subjects after adjusting for these parameters. Kempf et al. [16] also found that subjects with impaired fasting glucose have increased GDF-15 levels in the Xendos trial and they found high GDF-15 levels at baseline predict future diabetes risk. GDF-15 levels were also found to be elevated in patients with T2DM compared to healthy subjects [19]. Hong et al. reported higher GDF-15 levels in anemic patients with T2DM compared to those without anemia [23]. As the anemia may cause conflicting results also in impaired glucose tolerance group, we included only nonanemic subjects and found elevated GDF-15 levels in patients with impaired glucose tolerance. Our results suggest that increased GDF-15, as an anti-inflammatory peptide, may have a compensatory role in prediabetes, even in the early stages of subclinical inflammation.

The association between GDF-15 and hepcidin was first defined in hematologic diseases. GDF-15 levels were negatively correlated with hepcidin levels in chronic haemolytic anemia cases [14]. But some recent studies showed a bidirectional association between GDF-15 and hepcidin which was described as a positive correlation in elevated GDF-15 levels but a negative association in extremely elevated GDF-15 levels [31]. A positive correlation between GDF-15 and hepcidin levels was also reported in patients with T2DM including anemic subjects [23]. Our study demonstrated a significant positive relationship between GDF-15 and hepcidin levels suggesting persistent relationship between GDF-15 and hepcidin, even in nonanemic subjects.

GDF-15 levels were shown to be correlated with the parameters for abnormal glucose metabolism in some studies [16, 19, 21, 32]. Kempf et al. [16] found a positive correlation of GDF-15 with 2-h glucose but not with fasting glucose whereas other studies [19, 21, 32] reported a positive relationship between GDF-15 and fasting glucose. In our study, we found a positive correlation between GDF-15 levels and fasting glucose. In addition to fasting glucose, we calculated AUC-G and found its correlation with GDF-15 suggesting that both fasting and postprandial glucose may have impact on GDF-15 levels. A previous study has shown that high glucose induces GDF-15 expression and secretion in order to protect the cells from high glucose associated apoptosis in human umbilical vein endothelial cells [33]. The exact role of GDF-15 in the hyperglycemia seen in prediabetes needs to be clarified with further studies.

First limitation of our study is a relatively small sample size. Secondly, because of the cross-sectional design, we can not predict whether elevated GDF-15 levels lead to increased risk of incident diabetes.

In conclusion, impaired glucose tolerance is associated with increased GDF-15 levels even in the absence of anemia, but the levels of hepcidin are not significantly altered in prediabetic state. Further studies are needed to determine whether increase in GDF-15 concentration is a causal factor for the development of T2DM or only a compensatory mechanism in the deterioration of glucose metabolism.

## Figures and Tables

**Table 1:** Baseline characteristics of the patient and control groups.

	IGT (*n* = 37)	NGT (*n* = 32)	*p*
Age (years)	49.21 ± 10.72	47.45 ± 12.09	0.526
Gender (F/M)	24/13	21/11	0.999
BMI (kg/m^2^)	33.04 ± 7.75	31.06 ± 4.68	0.20
Hb (g/L)	14.14 ± 1.15	14.01 ± 1.10	0.617
Ferritin (ng/mL)	80.60 ± 76.50	66.81 ± 41.21	0.365
Fasting glucose (mg/dL)	106.0 [99.50–116.0]	92.0 [88.25–97.0]	**<0.001**
Fasting insulin (*μ*IU/mL)	14.92 ± 7.99	11.89 ± 6.64	0.109
HOMA-IR	3.95 ± 2.21	2.75 ± 1.49	**0.012**
AUC-G (mg/dL/min)	22002.97 ± 2917.88	14627.0 ± 2932.26	**<0.001**
Creatinine (mg/dL)	0.79 ± 0.20	0.74 ± 0.18	0.261
GFR (mL/min per 1.73 m^2^)	101.97 ± 27.42	107.70 ± 23.71	0.539
UA (mg/dL)	5.48 ± 1.55	4.84 ± 1.36	0.076
IL-6 (pg/mL)	1.24 [0.94–1.64]	1.07 [0.92–1.57]	0.270
hsCRP (mg/dL)	0.32 [0.15–0.73]	0.20 [0.11–0.48]	0.135
Hepcidin (ng/mL)	25.89 ± 15.60	26.94 ± 13.05	0.767
GDF-15 (pg/mL)	897.93 [691.57–1616.10]	770.36 [535.34–1040.04]	**0.026**

Normally distributed variables are presented as mean ± standard deviation and non-normally distributed variables are presented as median [25th, 75th percentile]. AUC-G: area under the curve for glucose, GFR: glomerular filtration rate, hsCRP: high sensitivity C-reactive protein, IGT: impaired glucose tolerance, NGT: normal glucose tolerance, BMI: body mass index, and UA: uric acid.

**Table 2:** Correlations between the demographic and laboratory values and the GDF-15/hepcidin levels.

	GDF-15		Hepcidin	
	*r*	*p*	*r*	*p*
Age	0.625	**<0.001**	0.237	0.052
BMI	−0.011	0.927	0.069	0.576
Fasting insulin	−0.034	0.789	0.153	0.223
Ferritin	0.128	0.294	0.449	**<0.001**
Hb	−0.018	0.881	0.135	0.270
UA	0.294	**0.015**	0.188	0.124
Creatinine	0.298	**0.018**	0.185	0.146
GFR	−0.393	**0.024**	−0.167	0.352
HOMA-IR	0.000	0.998	0.179	0.160
AUC-G	0.261	**0.033**	0.071	0.568
Fasting glucose	0.352	**0.004**	0.258	**0.035**
IL-6	0.151	0.216	−0.059	0.631
hsCRP	0.069	0.597	0.065	0.620
Hepcidin	0.248	**0.040**	—	—
